# Delayed migration of a Sapien 3 Ultra Resilia following transcatheter aortic valve implantation after selection of a smaller-sized valve

**DOI:** 10.1093/omcr/omae065

**Published:** 2024-07-09

**Authors:** Nagiko Mitsuoka, Tohru Takaseya, Ken-ichiro Sasaki, Kazuyoshi Takagi, Naoki Itaya, Kensuke Oshita, Masahiro Sasaki, Michiko Yokomizo, Yume Nohara, Hidefumi Kuroki, Yoshihiro Fukumoto, Eiki Tayama

**Affiliations:** Division of Cardiovascular Surgery, Department of Surgery, Kurume University School of Medicine, Asahi-machi 67, Kurume-si, Fukuoka 830-0011, Japan; Division of Cardiovascular Surgery, Department of Surgery, Kurume University School of Medicine, Asahi-machi 67, Kurume-si, Fukuoka 830-0011, Japan; Division of Cardiovascular Medicine, Department of Internal Medicine, Kurume University School of Medicine, Asahi-machi 67, Kurume-si, Fukuoka 830-0011, Japan; Division of Cardiovascular Surgery, Department of Surgery, Kurume University School of Medicine, Asahi-machi 67, Kurume-si, Fukuoka 830-0011, Japan; Division of Cardiovascular Medicine, Department of Internal Medicine, Kurume University School of Medicine, Asahi-machi 67, Kurume-si, Fukuoka 830-0011, Japan; Department of Anesthesiology, Kurume University School of Medicine, Asahi-machi 67, Kurume-si, Fukuoka 830-0011, Japan; Division of Cardiovascular Medicine, Department of Internal Medicine, Kurume University School of Medicine, Asahi-machi 67, Kurume-si, Fukuoka 830-0011, Japan; Department of Anesthesiology, Kurume University School of Medicine, Asahi-machi 67, Kurume-si, Fukuoka 830-0011, Japan; Division of Cardiovascular Medicine, Department of Internal Medicine, Kurume University School of Medicine, Asahi-machi 67, Kurume-si, Fukuoka 830-0011, Japan; Division of Radiology, Kurume University Hospital, Asahi-machi 67, Kurume-si, Fukuoka 830-0011, Japan; Division of Cardiovascular Medicine, Department of Internal Medicine, Kurume University School of Medicine, Asahi-machi 67, Kurume-si, Fukuoka 830-0011, Japan; Division of Cardiovascular Surgery, Department of Surgery, Kurume University School of Medicine, Asahi-machi 67, Kurume-si, Fukuoka 830-0011, Japan

**Keywords:** aortic valve implantation, migration, smaller size

## Abstract

This report discusses a rare case of delayed migration of a Sapien 3 Ultra Resilia (S3UR) valve following transcatheter aortic valve implantation. An 81-year-old Japanese woman had a borderline aortic annular size of 20–23 mm according to the manufacturer’s size chart. We chose to implant a smaller S3UR of 20 mm with an 80/20 depth ratio to allow for a second intervention, ensuring good hemodynamics and minimizing paravalvular leak. The patient initially had a favorable outcome despite an accidental 50/50 depth ratio during implantation. On postoperative day 3, the S3UR migrated into the left ventricular outflow tract. Emergency surgical aortic valve replacement was performed to retrieve the migrated valve. Use of the S3UR has led to a growing preference for smaller valve sizes. However, the risk of migration should be recognized. When an accidental 50/50 depth ratio implantation is encountered, post-dilation or second valve implantation should be performed immediately.

## Introduction

The Sapien 3 Ultra Resilia (S3UR) valve (Edwards Lifesciences Inc., Irvine, CA, USA) incorporates technical features that reduce the paravalvular leak (PVL) rate because of a revised outer sealing skirt and improve hemodynamic performance by enhancing valve opening. The benefits of the S3UR include the ability to select smaller-sized valves [1, 2]. This feature also offers advantages from the standpoint of second intervention.

## Case report

An 81-year-old Japanese woman was referred to our hospital with chest discomfort. An echocardiogram showed an aortic valve area of 0.82 cm^2^, a mean transaortic pressure gradient of 29 mmHg, and a peak aortic jet velocity of 3.8 m/s. Mild aortic and mitral regurgitation and moderate tricuspid regurgitation were observed. The left ventricular ejection fraction was 67% and the stroke volume index was 50 ml/m^2^. We diagnosed severe aortic stenosis based on her chest symptoms despite normal flow and a low gradient. The patient was at intermediate risk for complications if she underwent surgical aortic valve replacement (SAVR), with a Society for Thoracic Surgeons Predicted Risk of Operative Mortality score of 4.33%. After discussion within our heart team, in view of her advanced age and frailty, she was offered transfemoral transcatheter aortic valve implantation (TAVI) under conscious sedation.

**Figure 1:**
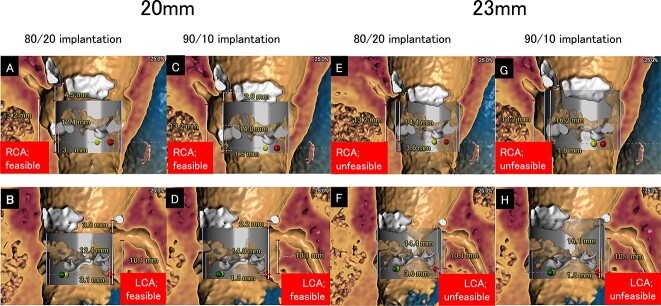
Simulation of S3UR implantation. (**A**) A 20-mm S3UR with 80/20 implantation allows future TAVI-in-TAVI in the RCA. (**B**) A 20-mm S3UR with 80/20 implantation allows future TAVI-in-TAVI in the LCA. (**C**) A 20-mm S3UR with 90/10 implantation allows future TAVI-in-TAVI in the RCA. (**D**) A 20-mm S3UR with 90/10 implantation allows future TAVI-in-TAVI in the LCA. (**E**) A 23-mm S3UR with 80/20 implantation does not allow future TAVI-in-TAVI in the RCA. (**F**) A 23-mm S3UR with 80/20 implantation does not allow future TAVI-in-TAVI-feasible in the LCA. (**G**) A 23-mm S3UR with 90/10 implantation does not allow future TAVI-in-TAVI in the RCA. (**H**) A 23-mm S3UR with 90/10 implantation does not allow future TAVI-in-TAVI in the LCA. LCA, left coronary artery; RCA, right coronary artery; S3UR, Sapien 3 Ultra Resilia, TAVI, transcatheter aortic valve implantation.

**Figure 2:**
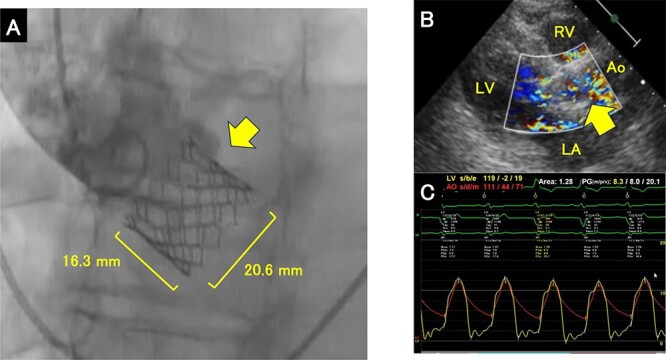
Images obtained after deployment of the THV. (**A**) Aortography shows 50/50 implantation as well as the height and diameter of the THV. The height is measured from the top to bottom of the stent. The diameter is measured from the right to left side of the stent. The arrow indicates the THV. (**B**) Transthoracic echocardiogram shows that the THV stayed in the aortic annulus. The arrow indicates the THV. (**C**) Pressure measurement at the aorta and left ventricle. THV; transcatheter heart valve, LA; left atrium, LV; left ventricle, Ao; Aorta.

We measured her aortic complex on preoperative computed tomography (CT) scans (Table 1). The aortic annular area was borderline, in the range of 20–23 mm on the S3UR manufacturer’s size chart. We simulated an implant position for both an 80/20 and a 90/10 ratio for sizes of 20 mm and 23 mm (Fig. 1). We chose a 20-mm S3UR and an implant position with an 80/20 ratio that allowed for a second intervention in the future in addition to good hemodynamics and less PVL. However, despite our best efforts, a 20-mm S3UR prosthesis was deployed in a ‘50/50’ position (Fig. 2A). Periprocedural transthoracic echocardiography and angiography demonstrated a favorable outcome immediately after implantation with no significant PVL or migration (Fig. 2B) during the TAVI procedure. Intraoperative imaging revealed acceptable positioning of the valve relative to the annulus with a mean valve gradient of 8 mmHg (Fig. 2C). We did not perform any additional procedures, such as post-dilatation or second valve implantation.

**Table 1:** Measurement of aortic complex by multidetector computed tomography

		Measurer 1	Measurer 2	Measurer 3	Measurer 4
Annulus diameter	short/long (mm)	18.7/23.4	18.3/21.6	17.4/22.7	19.1/24.3
Annulus	Perimeter (mm)	67.4	64.2	64.4	66.6
Annulus	Area (mm^2^)	347	312	319	339
LVOT	Perimeter (mm)	68.6	67.0	65.9	67.2
LVOT	Area (mm^2^)	352	336	327	337
STJ diameter	short/long (mm)	22.9/23.2	21.1/22.0	19.4/22.3	21.1/21.3
SOV diameter	(RCC) (mm)	26.4	25.1	26.6	26.0
SOV diameter	(LCC) (mm)	27.7	25.4	26.4	25.3
SOV diameter	(NCC) (mm)	25.7	26.3	27.5	27.3
RCA height (mm)		13.6	15.0	13.2	14.7
LCA height (mm)		11.0	12.4	10.1	10.7

LCA, left coronary artery; LCC, left coronary cusp; LVOT, left ventricular outflow tract; NCC, non-coronary cusp; RCA, right coronary artery; RCC, right coronary cusp; SOV, sinus of Valsalva; STJ, sinotubular junction.

**Figure 3:**
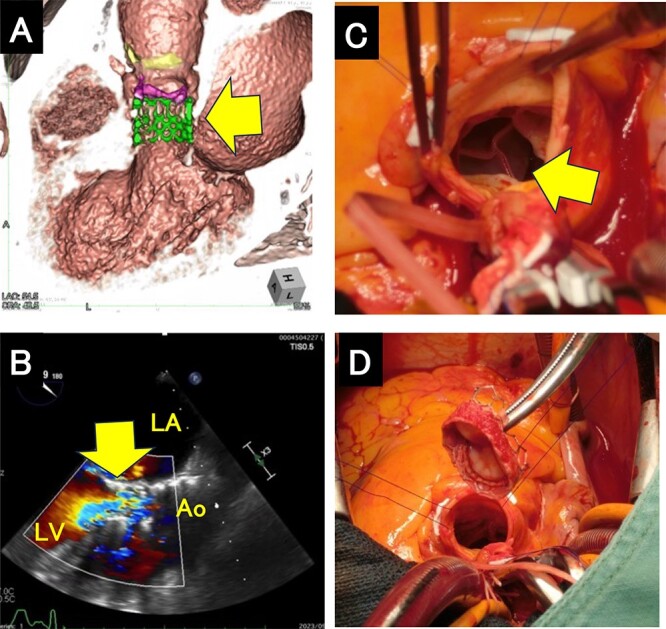
Migration of the THV. (**A**) Computed tomography scan showing that the THV had migrated into the left ventricular outflow tract. The arrow indicates migrated THV. (**B**) Transesophageal echocardiogram obtained under general anesthesia showing severe paravalvular leak. The arrow indicates migrated THV in LVOT (**C**) The upper stent of the THV located under the aortic valve. The arrow indicates migrated THV (**D**) The explanted THV. THV; transcatheter heart valve, LVOT; left ventricular outflow, LA; left atrium, LV; left ventricle, Ao; Aorta.

The patient mobilized on postoperative day 1. However, a chest radiograph on postoperative day 3 revealed congestion. Physical examination was remarkable for a diastolic murmur heard best at the lower left sternal border. CT revealed migration of the S3UR into the left ventricular outflow tract (LVOT) (Fig. 3A). The patient subsequently underwent open heart surgery. Transesophageal echocardiography after general anesthesia showed that the S3UR had migrated into the LVOT, causing massive PVL, and had also contacted the anterior mitral leaflet, with accompanying signs of mitral valve dysfunction (Fig. 3B). Intraoperative examination showed that the S3UR had dislodged from the aortic valve to the left ventricular side (Fig. 3C). We performed SAVR using a Perceval S valve (LivaNova, London, UK) after retrieving the migrated valve (Fig. 3D). The patient was discharged 20 days following SAVR and recovered without complications.

## Discussion

There have been reports of migration as a complication of TAVI [3–5]. To the best of our knowledge, this is the first report of delayed migration of an S3UR valve. Kim et al. present the largest series to date of TAVI valve migration patients from a cohort of over 29 000 TAVI cases. The rate of TAVI valve migration was approximately 1% and was associated with high morbidity and mortality. The use of self-expanding or first-generation prostheses and presence of bicuspid aortic valve were independent predictors for the onset of TAVI valve migration [5]. Additionally, Kim et al. defined the time for the time for TAVI valve migration. The migration in our case might be occurred between the postoperative day 1and 3, so we defied delayed migration [5]. The main cause of migration in our case was placement of the S3UR at a lower position. However, the migration might also have been influenced by selection of a smaller-sized S3UR. Our case had a borderline-sized aortic annulus of 20–23 mm. The choice of S3UR size is particularly important in patients with a borderline S3UR size measured on preoperative CT of the aortic valve orifice area. There are some reports on selection of transcatheter heart valve (THV) size for a borderline-sized aortic annulus [6, 7]; the options are to over-expand a smaller-sized THV or under-expand a larger-sized THV. There are some advantages if a smaller-sized THV is selected, including a lower risk of aortic root injury, which improves the potential feasibility of repeat TAVI in the future because of the low risk of sinus sequestration and left main coronary obstruction. However, there are also some disadvantages, in that overexpanding a smaller-sized THV may increase the risks of suboptimal hemodynamic performance and worse PVL. The S3UR valve, released in 2023, is the latest iteration of the balloon-expandable Edwards valve family in Japan. It incorporates technical features aimed at simplifying TAVI procedures, as well as further reducing the PVL rate through a revised outer sealing skirt, which has an approximately 40% increase in height and is designed to allow up to 50% more surface contact area with the native valve anatomy for improved annular sealing [1]. There is a report suggesting that the hemodynamic performance of the S3UR is superior to that of the S3 when considering small-sized valves [2]. We selected a smaller-sized (20-mm) S3UR in the expectation that the S3UR with its high skirt could reduce PVL and provide good hemodynamics because of enhanced valve opening.

We originally intended S3UR implantation with 80/20 ratio positioning but accidentally implanted it deep with 50/50 positioning. We did not perform any additional procedure or post-dilatation and did not implant a second valve because there was no PVL and hemodynamics were good. Unfortunately, the S3UR migrated into the LVOT on day 3 after TAVI. A 50/50 depth ratio during implantation may indicate the highest likelihood of valve instability because of the morphology during full expansion of the S3UR, which is characterized by a narrowing waist [8] (i.e. the center of the S3UR is the narrowest, resembling an hourglass). We should have added post-dilatation or implanted a second valve despite the lack of PVL and the good hemodynamics. These additional interventions might have prevented delayed migration of the valve.

Use of the S3UR has led to a growing preference for smaller-sized valves, presenting advantages in terms of second intervention. Nevertheless, it is essential to recognize the risk of migration. When a 50/50 depth ratio implantation is encountered, post-dilation or implantation of a second valve should be considered.

## Data Availability

Data are available on request from the corresponding authors.
